# Government attention allocation in promoting strategic emerging industries in China’s less-developed central region: A policy text analysis of Shanxi province

**DOI:** 10.1371/journal.pone.0347000

**Published:** 2026-06-24

**Authors:** Xia Li

**Affiliations:** School of Education Science, Shanxi Normal University, Taiyuan, Shanxi, China; Swiss Federal Technology Institute of Lausanne, SWITZERLAND

## Abstract

Government attention is recognized as a crucial factor in facilitating the sustainable and leapfrog development of strategic emerging industries (SEIs) in less-developed central regions. Focusing on Shanxi province, a typical less-developed central region in China, this study investigates the allocation logic of government attention by analyzing 60 policy documents from 2010 to 2025. Drawing on the attention-based view and policy instrument theory, we establish an Actor-Instrument-Theme framework, and employ a mixed-methods approach combining case study, Latent Dirichlet Allocation (LDA) and Social Network Analysis (SNA). The results reveal weak collaboration among policy actors, an over-reliance on supply-side and environmental policy instruments, and a pyramid-shaped thematic priority structure. This pattern is defined as a foundation-first, gradient-layout approach. While this model may be efficient for early-stage industrial takeoff, it risks long-term distortions such as innovation crowding-out, market entry barriers, and path-dependence lock-in. To promote the sustainable development of SEIs, it is recommended that local governments strengthen policy coordination, optimize policy instrument portfolios, and ensure a balanced deployment of industrial resources.

## 1. Introduction

Amidst the new global wave of technological revolution and industrial transformation, cultivating and developing strategic emerging industries (SEIs) has become a strategic choice for nations to secure future competitive advantages. For China, promoting the coordinated regional development of SEIs is an intrinsic requirement for building a modern industrial system and a critical pathway to addressing unbalanced and inadequate development. Developed eastern regions are capital-intensive and innovation-driven. By contrast, China’s less-developed central regions face inherent disadvantages and compounding structural challenges. These include difficulties in capital accumulation, technological innovation, and talent attraction, constraints that lock them into the structural dilemma of the resource curse. Against this, governments in less-developed central regions, as key suppliers and market shapers of the regional industrial ecosystem, play a pivotal role through their decision-making cognition and resource allocation directions. Government attention, as a scarce cognitive and administrative resource, dictates policy agenda priorities, public resource flows, and the selection and configuration of policy instrument portfolios, thereby becoming a core variable influencing the effectiveness of regional industrial transformation. Specifically, government attention refers to the focus placed on particular issues when processing complex information. Under tight resource constraints, the allocation of attention by governments in less-developed central regions is particularly critical, profoundly impacting policy implementation efficacy and market expectation shaping. Accordingly, this study poses the following research questions: In the process of promoting SEI development, how is government attention allocated in less-developed central regions? What structural characteristics does this allocation exhibit?

Existing research on this topic can be primarily categorized into two dimensions: spatial scope and research content. Regarding spatial scope, studies have either concentrated on developed eastern regions or the national macro-level. For instance, Zhai et al. took Nanjing as a case to explore the spatial interaction mechanisms between the geography and network agglomeration of SEIs [1]. Conversely, Xu et al. analyzed the regional disparities and spatial convergence characteristics of collaborative innovation in SEIs based on panel data from 31 provinces [2]. Extending beyond the Chinese context, international scholarship has long scrutinized the spatial dynamics of emerging industries. Seminal research on the agglomeration of high-tech industries in Silicon Valley [3] and the spatial clustering of biotechnology in Germany [4] has elucidated how regional innovation systems shape industrial specialization. More recently, analyses of Germany’s Industrie 4.0 initiative and the Advanced Manufacturing Partnership in the United States have underscored the critical role of regional policy in cultivating emerging industrial clusters [5]. Regarding research content, the existing literature can be broadly categorized into three strands. The first focuses on the spatial layout of SEIs, such as the analysis of spatial evolutionary characteristics in the Beijing-Tianjin-Hebei region by Ji et al. [6] and the exploration of industrial co-agglomeration patterns in the Yangtze River Delta by Zhao et al. [7]. The second strand involves the analysis of the SEI policy system. From a policy instrument perspective, Yao and Hu analyzed SEI plans and specialized policies across 31 provinces, finding a relatively high degree of overall policy comprehensiveness [8]. Internationally, Japan’s strategic deployment of tax incentives, low-interest loans, and technology demonstration projects within its robotics sector illustrates how advanced economies calibrate instrument portfolios to mitigate market uncertainty [9]. From a policy theme perspective, Xie and Wang examined the evolution of China’s 5G SEI policies [10]. The third strand concerns the government’s role in facilitating SEI development. Through empirical research, Tang and Qiu analyzed the leading and market-coordinating roles of government-guided funds in SEI development [11]. Yao et al. investigated how artificial intelligence can boost the formation of new quality productive forces within SEIs [12]. Theoretically, the international debate on the state’s role in shaping emerging industries remains highly prominent. Mazzucato posits that the state functions as an “entrepreneurial investor” that assumes substantial risks in early-stage technologies, a concept exemplified by the U.S. Defense Advanced Research Projects Agency and the National Nanotechnology Initiative [13]. Furthermore, policymaking in advanced economies frequently incorporates structured mechanisms of “policy learning”, for instance, Germany’s Fraunhofer Institutes systematically evaluate technology policies and recalibrate instruments based on performance metrics [14].

Although existing research has made significant contributions, it still suffers from several limitations. First, there is a regional imbalance in research focus. Most studies are situated in developed regions or at the national macro-level, paying insufficient attention to the structural constraints faced by less-developed central regions—such as resource endowments, institutional environments, and market development levels, thereby overlooking their critical role as strategic pivots for coordinated regional development. Second, the research perspective is often singular. Many studies analyze the issue from a single lens, such as policy instruments or themes, without adequately considering the multidimensional characteristics of SEI policies, including fundamental features and implementation priorities. Third, theoretical depth remains insufficient. Most studies treat the government as an exogenous intervention variable, lacking in-depth analysis of its internal decision-making and cognitive mechanisms. As material carriers of government decision-making philosophy and governance priorities, policy documents provide a reliable basis for observing government attention [15]. Addressing the aforementioned theoretical gaps, this study makes three primary contributions to theory building. First, by applying the Attention-Based View (ABV) to policy text analysis, it operationalizes government attention into three measurable dimensions: policy actor, policy instrument, and policy theme. Second, it proposes an Actor–Tool–Theme analytical framework that captures the structural interplay among these dimensions, thereby transcending the limitations of single-dimension studies. Third, it inductively constructs the concept of a the “foundation-first, gradient-layout” as a context-specific ideal type for resource-dependent, less-developed central regions, which serves as a hypothesis-generating foundation for future comparative and causal research.

Drawing upon attention allocation and policy instrument theories, this study takes Shanxi province as a case to systematically examine the structure of government attention allocation in China’s less-developed central regions during the promotion of SEIs. Employing a mixed-methods approach integrating Latent Dirichlet Allocation (LDA) topic modeling and Social Network Analysis (SNA), this research aims to provide a theoretical basis for optimizing the industrial policy system, fostering industrial structure upgrading, and transforming the development model in these regions, thereby facilitating high-quality regional leapfrog development. The remainder of this paper is structured as follows: Section 2 reviews the relevant literature. Section 3 describes the research design. Section 4 presents and analyzes the empirical results. Finally, Section 5 discusses the findings, concludes the study, and offers policy recommendations.

## 2. Theory and analytical framework

### 2.1. Theory

Government attention allocation is pivotal in shaping SEI development. Originating in the field of psychology, the concept of attention was introduced to management science by Herbert Simon in *Administrative Behavior*, which posits that organizational management and decision-making are constrained by bounded rationality and attention scarcity [16]. Subsequently, this concept was extended to political science and public policy, where scholars have examined how governments selectively attend to policy problems and how government attention allocation shapes agenda setting [17]. In the context of government-promoted SEI development, government attention refers to the cognitive and behavioral process through which the government identifies, encodes, interprets, and focuses on SEI solutions [18]. Based on ABV, government attention, conceptualized as a scarce administrative resource, constitutes a critical intermediary link in governmental information processing and policymaking. The direction, intensity, and persistence of government attention allocation directly influence SEI policy orientation, resource distribution, and development pace, potentially leading to path divergence and performance variations in industrial development [19]. Consequently, the logic of government attention allocation affects the design and implementation efficacy of the SEI policy system, ultimately determining the institutional authority and resource scale that SEI issues can secure within the regional governance structure. This impact is particularly significant for China’s less-developed central regions. Facing tight resource constraints, weak market mechanisms, and a thin industrial base, government attention allocation in promoting SEI development directly determines the priority and support level of industrial policies. At a deeper level, it influences whether the region can overcome development bottlenecks and achieve industrial structure upgrading and economic catch-up. When optimally allocated, government attention in these regions can effectively compensate for market failures, precisely guide limited resources to key sectors, and catalyze industrial ecosystem formation. Conversely, misdirected or fragmented attention allocation may lead to policy idling, resource dissipation, and the loss of strategic opportunities. Thus, understanding and optimizing the logic of government attention allocation constitutes a critical governance issue for less-developed central regions to cultivate emerging industries and achieve high-quality regional development.

### 2.2. Analytical framework

**Fig 1:**
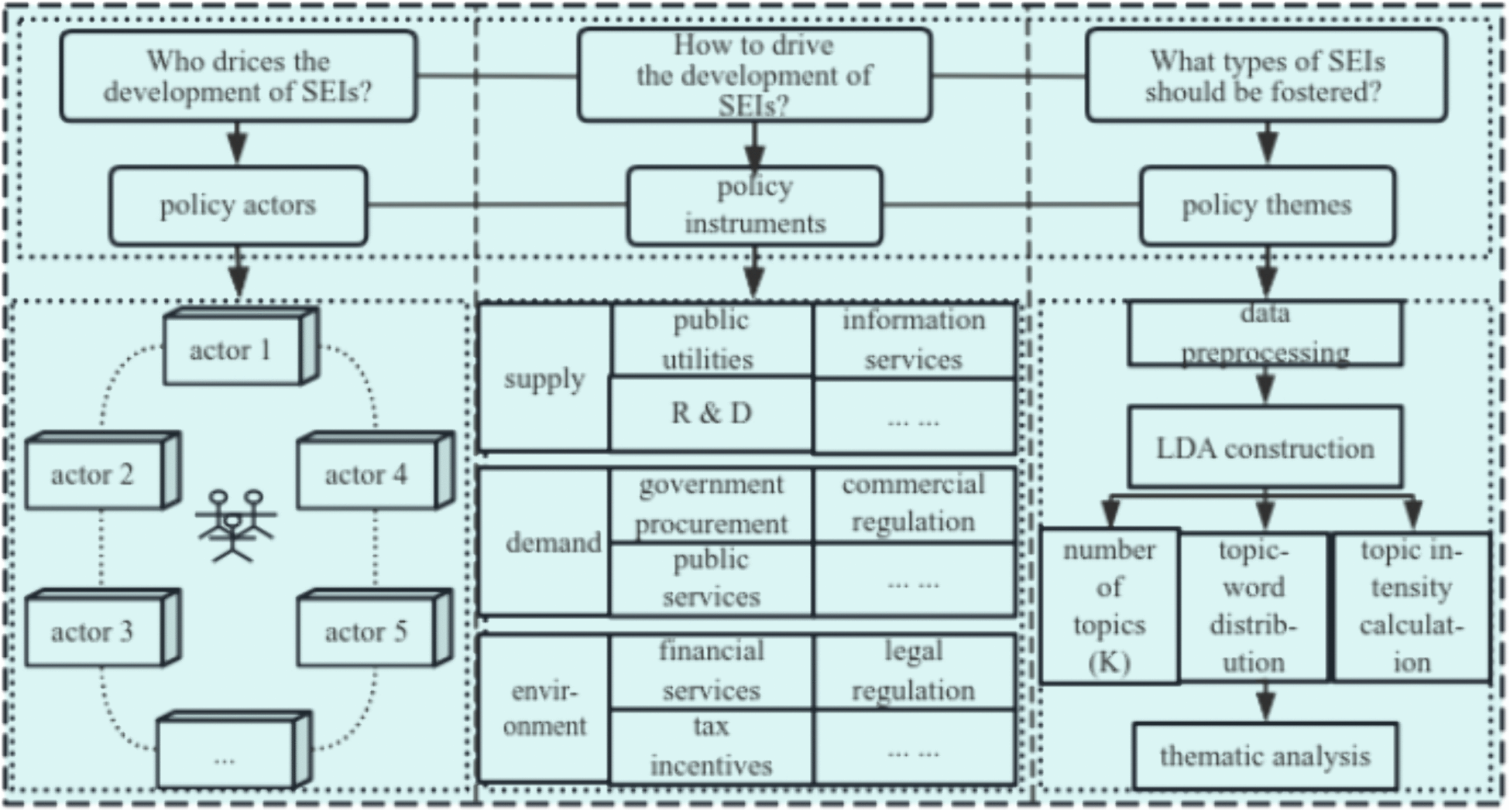
Analytical framework. (Source: Compiled by the author).

Government attention allocation constitutes the policy decision-making process of government actors, characterized by the selection of specific themes, content, and instruments. The question of how local governments allocate attention to promote SEI development can be deconstructed into three sub-questions: who promotes SEI development, how this development is promoted, and which specific SEIs are promoted. Accordingly, by mapping these three sub-questions onto policy texts, this study constructs a three-dimensional analytical framework of Policy Actor–Policy Instrument–Policy Theme (Fig 1).

Policy actors refer to the issuing bodies of SEI policies and constitute core elements of the policy system, playing vital roles in stages including policy formulation, implementation, supervision, and evaluation. Given the scope and development requirements of SEIs, policy actors typically involve multiple government departments. These actors primarily institutionalize the goals and measures for SEI development through the formulation and issuance of policy documents. Therefore, analyzing policy actors facilitates an understanding of the driving forces and organizational structures for advancing SEIs. This analysis can focus on the distributional characteristics and collaborative networks of policy actors. Policy instruments are the means and mechanisms adopted by the government to promote SEI development and represent a core dimension of policy text analysis. Analyzing policy instruments reveals the approaches and pathways the government employs to advance SEIs. Based on the research of Rothwell and Zegveld [20], policy instruments are classified according to their content attributes in SEI policy texts, into supply-side, environmental, and demand-side instruments. This typological framework has been widely adopted and extended in international scholarship; notably, Rogge and Reichardt [21] adapted it to dissect policy mixes for sustainability transitions. Supply-side policy instruments refer to the methods the government uses to promote SEI development from the production side, such as support for public services, technology, infrastructure, human resources, and capital investment, which directly expand and optimize factor supplies. Demand-side policy instruments refer to the methods used to promote SEI development from the consumption side, such as streamlining administration, delegating power, opening up to the outside world, and government procurement. These instruments reduce market uncertainty while actively exploring and stabilizing the demand market for industrial development. Environmental policy instruments refer to institutionalized efforts by the government to indirectly influence SEIs from the environmental dimension, such as improving government regulation, fiscal and tax policies, financial policies, legal frameworks, and target planning. Policy themes constitute a crucial component of SEI policy texts and represent the core issues in advancing SEI development. Through topic mining, the analysis of policy themes can extract the government’s focal points and areas of emphasis in promoting SEI development, thereby revealing the local government’s strategic allocation approach. Regarding specific analytical strategies, software is typically utilized to identify and extract topic words from a large text sample, allowing for an in-depth analysis of policy texts to reflect the government focus on SEI development in less-developed central regions.

## 3. Research design

### 3.1. Method

This study employs a case study approach to investigate the government attention allocation mechanism in less-developed central regions during SEI policy formulation. The case study approach captures government behavior and its underlying logic within a real-world context, enabling process tracing and mechanism identification for complex policy phenomena. Unlike multiple-case studies, which often prioritize external validity, the single-case study aims to conduct an in-depth analysis of a theoretically representative or contextually typical case, thereby generating contextualized and mechanistic theoretical insights [22,23]. Internationally, the single-case study has been recognized as a valuable strategy for theory building, particularly in under-researched contexts [24].

For data analysis, this study integrates LDA and SNA to analyze SEI policy data. The former identifies and summarizes policy themes from a thematic dimension, while the latter analyzes policy-issuing bodies from the policy actor dimension. LDA is an unsupervised probabilistic machine learning method designed to mine text data, identify latent topics, and facilitate their visualization. Originally proposed by Blei, Ng, and Jordan in 2003 [25], LDA is now widely applied in the text analysis and data mining of policy documents, patents, online reviews, and academic literature. Compared to traditional text analysis methods, LDA is not constrained by chapter or paragraph divisions; it efficiently extracts underlying semantic structures, offering advantages in objectivity, reproducibility, and efficiency. SNA is rooted in statistical theory, graph theory, and social network theory. By treating network relationships as the basic unit of analysis, SNA quantitatively analyzes network nodes and ties to construct and visualize social networks, thereby revealing the structural properties, collective behaviors, and interactive dynamics within the SEI policy actor network [26]. SNA is widely applied across disciplines such as management, sociology, and education.

### 3.2. Case selection

Guided by the case study principles of contextual embeddedness and problem typicality, and considering regional economic development levels and industrial structures, this study selects Shanxi province as the research case. From an economic development perspective, Shanxi province aligns with the criteria for a less-developed central region. As defined in the *Several Opinions of the CPC Central Committee and the State Council on Promoting the Rise of the Central Region* and supported by existing research [27,28], Shanxi’s per capita GDP has long been relatively low, positioning it at a disadvantage in the regional development pattern. Internationally,scholars have emphasized that resource-dependent regions face specific path‑dependency challenges that shape government policy attention [29]. As shown in Table 1, Shanxi’s five-year average per capita GDP is significantly below that of leading provinces like Hubei and Hunan, and places it in the lower tier of the central region. From an industrial structure perspective, Shanxi is a traditional resource-based region, making it a representative case of less-developed central areas. As China’s only provincial-level pilot zone for comprehensive resource-based economic transformation, Shanxi has long been dependent on a “coal-dominated” industrial structure. This reliance has resulted in economic vulnerability and profoundly shaped the government’s behavioral logic and cognitive pathways. This extensive growth model, driven by high input, high consumption, and high emissions, makes SEI development a strategic imperative and a critical breakthrough for Shanxi to overcome structural bottlenecks and achieve sustainable development.

**Table 1:** Per capita GDP of six central provinces (2019-2023).

Region	Province	Per capita GDP (RMB)					5-year average (RMB)
		2019	2020	2021	2022	2023	
Less-developed central region	Shanxi	45724.0	50528.0	64821.0	73675.0	73984.0	61746.4
	Anhui	58496.0	63426.0	70321.0	73603.0	76830.0	68535.2
	Jiangxi	53164.0	56871.0	65560.0	70923.0	71216.0	63546.8
	Henan	56388.0	55435.0	59410.0	62106.0	60073.0	58682.4
	Hubei	77387.0	74440.0	86416.0	92059.0	95538.0	85168.0
	Hunan	57540.0	62900.0	69440.0	73598.0	75938.0	67883.2

Source: Data are sourced from *China Statistical Yearbook*.

### 3.3. Policy text selection

SEI policy texts are the primary carriers of government attention allocation. This study selects SEI policies as the research sample, ultimately identifying 60 policy texts from 2010 to 2025 (Table 2). To ensure sample representativeness and rigor, the PKULaw Legal Database and the China Legal Knowledge Resource Database were utilized as primary data sources. Additionally, to ensure comprehensiveness, data were supplemented from official websites of the provincial governments, the Departments of Science and Technology, the Development and Reform Commissions, and other relevant bodies. Detailed data collection procedures are presented in Table 3. Keyword-based retrieval is considered more systematic than categorical collection [30]. Thus, the retrieval strategy employed “strategic emerging industries” as the search term, limited to titles. Regarding document type, informal documents such as approvals, letters, and announcements were excluded; the focus was restricted to formal policy documents, including plans, notices, measures, guidelines, and programs. The retrieval period spanned from 2010 to 2025, starting from the promulgation of the *Decision of the State Council on Accelerating the Cultivation and Development of Strategic Emerging Industries* in October 2010, which marked the formal establishment of SEIs as a national strategy. Regarding issuing authorities, samples were restricted to provincial-level documents. Based on this strategy, the initial search yielded 1,453 policy documents. After manually removing 1,393 irrelevant, duplicated, or expired documents, a final sample of 60 valid texts was obtained. The specific criteria for valid policy samples were as follows:

**Table 2:** Summary of SEI policies (Partial).

No.	Title	Document No.	Issuing body	Date issued
P1	Opinions of the Shanxi Provincial People’s Government on Accelerating the Cultivation and Development of Strategic Emerging Industries	Jin Zheng Fa [2011] No. 21	Shanxi Provincial People’s Government	2011.07.28
P2	Notice on the Implementation Plan for the Cultivation Project of Leading Talents in Emerging Industries in Shanxi Province	Jin Ren She Ting Fa [2012] No. 40	Shanxi Provincial Department of Human Resources and Social Security	2012.04.11
P3	Opinions on Further Promoting the Standardized Development of the Financing Guarantee Industry in the Province	Jin Zheng Ban Fa [2012] No. 35	Shanxi Provincial People’s Government	2012.05.21
P4	Several Opinions on Maintaining Stable and Relatively Fast Economic Growth	Jin Zheng Fa [2012] No. 21	Shanxi Provincial People’s Government	2012.06.11
P5	Notice on the “Twelfth Five-Year Plan” for the Development of Strategic Emerging Industries in Shanxi Province	Jin Fa Gui Hua Fa [2013] No. 1153	Shanxi Provincial Development and Reform Commission	2013.06.14
…	…	…	…	…
P56	Notice on Organizing the Declaration of Provincial Strategic Emerging Industry Integration Clusters	Jin Fa Gao Xin Fa [2023] No. 67	Shanxi Provincial Development and Reform Commission	2023.03.06
P57	Notice on the Support Catalog for Strategic Emerging Industries (for Trial Implementation)	Jin Gong Xin Yun Xing Gui Zi [2023] No. 1	Shanxi Provincial Department of Industry and Information Technology, Shanxi Provincial Development and Reform Commission, Shanxi Provincial Department of Ecology and Environment, Shanxi Provincial Energy Bureau	2023.05.15
P58	Measures for the Accreditation and Management of Provincial New-type R&D Institutions	Jin Ke Gui [2024] No. 4	Shanxi Provincial Department of Science and Technology	2024.02.07
P59	Notice on Several Measures of Shanxi Province to Promote the Integrated Development of Advanced Computing Power and Artificial Intelligence	Jin Zheng Ban Fa [2024] No. 35	Shanxi Provincial People’s Government	2024.06.25
P60	Notice on the Management Measures for the Special Fund for the Development of the Provincial Private Economy	Jin Cai Gui Jian [2025] No. 1	Shanxi Provincial Department of Finance, Shanxi Provincial Bureau of Private Economy Development	2025.04.29

**Table 3:** Data details of SEI policies.

Retrieval criteria	Retrieval content
Time span	2010–2025
Data sources	PKULaw Legal Database, Official websites of provincial government departments (supplementary)
Search terms	“Strategic Emerging Industries”, “Strategic Emerging Industry Policies”
Search type	Title Search
Search query	Title = “Strategic Emerging Industries” + “Strategic Emerging Industry Policies”
Date of retrieval	June 8, 2025
Retrieval results	60 valid policy documents

(1) Relevance: The document must be closely related to SEI development, directly reflecting governmental attitudes and measures.
(2) Scope: The document must encompass various aspects of SEI development.
(3) Document type: The document must be a provincial-level policy, such as a notice, opinion, measure, or decision.
(4) Validity: Documents explicitly superseded by newer policies or those with expired dates were excluded.
(5) Uniqueness: Identical documents retrieved from different databases were merged.

### 3.4. Research process

#### 3.4.1. SNA-based research process.

**Fig 2:**
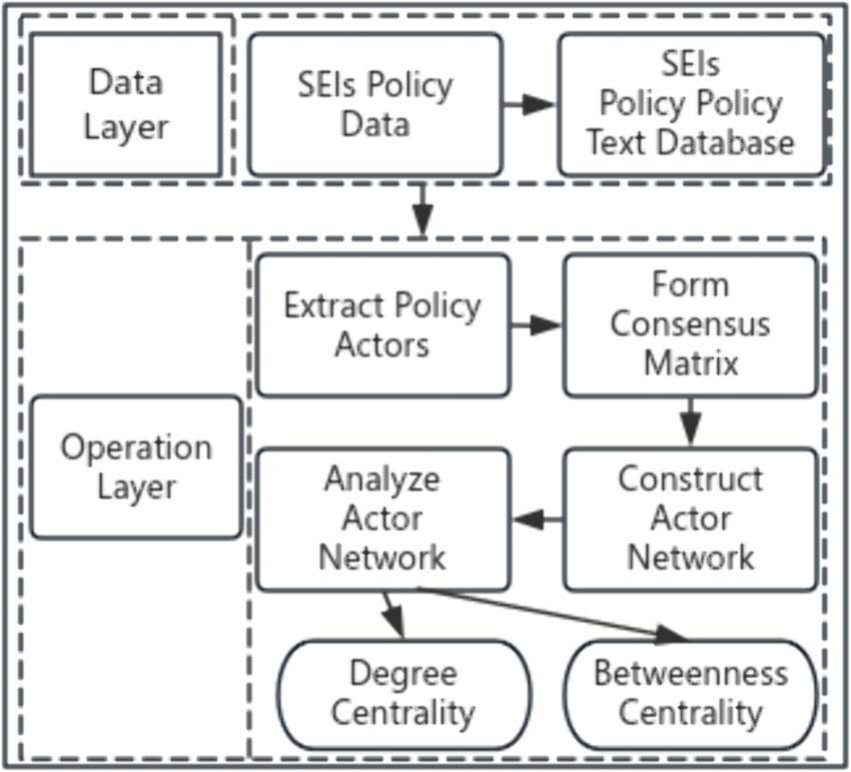
Social network analysis procedure (Source: Original figure created by the author).

This study utilizes Gephi to analyze the collaborative networks among SEI policy actors. The procedure is illustrated in Fig 2 and detailed below. First, policy actors are extracted from the 60 SEI policy documents. Second, a co-occurrence matrix is constructed. An undirected edge connects any two actors that jointly issued a policy; for each additional co-issuance by the same pair, the edge weight increases by one. Policies issued by a single actor are excluded to focus on inter-organizational collaboration rather than unilateral actions. Third, the policy actor network is generated in Gephi, with nodes representing actors and edges representing collaborative ties. Fourth, network metrics are calculated. Degree centrality quantifies an actor’s direct connections; a high degree indicates a central collaborator, whereas a low degree suggests limited collaboration. Betweenness centrality measures the frequency with which an actor lies on the shortest path between two others, indicating its brokerage or bridging role [31]. Fifth, a sensitivity analysis is conducted by removing isolated nodes and recalculating the metrics. The top-five rankings for both degree and betweenness centrality remain unchanged, with the Shanxi Provincial Development and Reform Commission remaining the most central actor. This confirms that the primary conclusions are robust to the inclusion or exclusion of peripheral actors.

#### 3.4.2. LDA-based research process.

**Fig 3:**
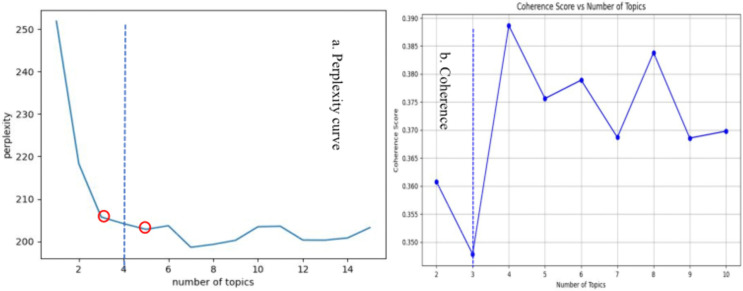
Perplexity curve (a) and coherence curve (b) (Source: Generated from the author using Python).

This study implements the LDA topic modeling process using Python 3.12 through the following steps. First, the policy texts were preprocessed. For word segmentation, the jieba library was utilized for its robust performance in part-of-speech tagging, keyword extraction, and Simhash similarity computation. For stop words, comprehensive stop-word lists from Baidu, the Harbin Institute of Technology, and the Sichuan University Artificial Intelligence Laboratory were integrated. Additional preprocessing included removing numeric values and punctuation, excluding words shorter than two Chinese characters, and filtering terms appearing in fewer than three documents to reduce noise. A document-term matrix was constructed using a bag-of-words representation with term frequency weighting. Second, we determined the number of topics. Determining the optimal number of topics (K) is a prerequisite for generating objective and scientifically valid topics. Drawing on existing research [32], the Gensim library was utilized to calculate perplexity and coherence scores to determine the optimal K. Excessively high topic counts can artificially lower perplexity, potentially causing overfitting and generating meaningless topics; conversely, excessively low topic counts yield high perplexity, resulting in overly broad topics [33,34]. Accordingly, the optimal K follows the principle of low perplexity with a moderate topic count [25]. Perplexity measures the model’s predictive uncertainty on unseen data; although lower values generally indicate a better fit, excessively low values suggest the model is memorizing noise rather than detecting genuine underlying patterns. Coherence reflects the semantic similarity among keywords within a topic, showing a positive correlation with topic division effectiveness [35]; higher scores denote greater interpretability. Both metrics were calculated using Equations (1) and (2) (Table 4) and plotted to identify the optimal K (Fig 3). As shown in Fig 3, the perplexity curve begins to plateau at K = 4, where the coherence score also peaks; thus, K = 4 was determined as the optimal number of topics. To ensure reproducibility, a random seed (random_state = 42) was set for all LDA iterations. Third, the LDA model was trained. With K set to 4, latent topics were extracted from the segmented texts using Python’s scikit-learn library, incorporating the top 1,000 feature words for model training. Fourth, visualization and topic intensity analysis were conducted. Guided by the fundamental attributes of SEI governance, the thematic focus of local government attention was extracted and summarized under the optimal topic structure. Fifth, topic labeling and robustness checks were performed. For each topic, the 15 highest-probability terms were extracted based on a relevance (λ = 0.6), and labels were inductively assigned based on semantic coherence. Two independent researchers reviewed the term lists, resolving disagreements through discussion. Robustness was assessed by re-estimating the model with K = 3 and K = 5. The coherence scores for K = 3, 4, and 5 were 0.343, 0.388, and 0.376, respectively. At K = 5, the “intelligent upgrading” topic fragmented into two distinct sub-themes; at K = 3, “institutional reform” merged into “talent echelon construction.” Hence, the primary findings demonstrate structural stability.

**Table 4:** Perplexity, coherence, and model parameters.

Type	Formula	Equation No.
Perplexity calculation formula	$\text{Perplexity}(D)=\text{exp}\left\{-\frac{\sum_{d=\text{1}}^{M}\text{log}p({𝐰}_{d})}{\sum_{d=\text{1}}^{M}N_{d}}\right\}$	Equation (1)
Coherence calculation formula	$C_{\text{UMass}}=\frac{\text{2}}{N(N-\text{1})}\sum_{i=\text{1}}^{N}\sum_{j=i+\text{1}}^{N}\text{log}\frac{P(w_{i},w_{j})+\in }{P(w_{j})}$	Equation (2)
LDA parameters	max_features: 1000, learning_offset: 50, max_iter: 50, n_components: 4, max_df: 0.9, min_df: 5	

#### 3.4.3. Coding procedure for policy instrument classification.

The classification of policy instruments into supply-side, environmental-side, and demand-side categories was conducted through a systematic manual coding procedure. First, the coding unit was determined. Each policy document was segmented into individual policy measures, defined as specific clauses or paragraphs articulating a distinct government action or support mechanism. Second, the coding scheme was formulated. The three instrument types were operationalized based on Rothwell and Zegveld [20] with context-specific adaptations. Supply-side policy instruments directly provide or upgrade production factors, including financial funding, talent training, R&D support, infrastructure construction, public services, and platform or carrier development. Environmental policy instruments shape the external institutional context, encompassing strategic planning, fiscal and tax incentives, financial policies, regulatory controls, target setting, and intellectual property protection. Demand-side policy instruments stimulate market demand, including government procurement, application demonstrations, consumer subsidies, market expansion, and trade facilitation. Third, the coding process was executed. Two independent coders, both public policy doctoral researchers, manually read all 60 policy documents. Each policy measure was assigned to one of the three categories or excluded as if irrelevant. The coders were trained on ten sample policies excluded from the final dataset to ensure consistent interpretation of the coding scheme. Fourth, reliability was assessed. Inter-coder agreement was assessed on a randomly selected subset of 20 policies (33.3% of the sample). The initial agreement rate was 86.2%, with a Cohen’s kappa coefficient of 0.81, indicating substantial agreement. Disagreements were resolved through discussion. The remaining policies were coded by one coder, with ambiguous cases reviewed by the second. The final classification counts are based on the consensus results.

## 4. Results

### 4.1. Analysis of SEI policy actors

#### 4.1.1. Analysis of policy-issuing by actors.

High-frequency policy actors were extracted from the SEI policy texts and are listed in Table 5. Between 2010 and 2025, a total of 65 actors participated in the formulation and issuance of SEI policies, including restructured or dissolved departments, indicating the diversity of China’s SEI decision-making bodies. The Shanxi Provincial People’s Government ranked first, participating in 33 policy issuances, 27 of which were independent. Textual data shows that 43 institutions issued policies independently, constituting 71.67% of all issuing bodies. Among these independent issuances, 55.81% originated from the SPPG. Additionally, the Shanxi Provincial Committee of the Communist Party of China, the Shanxi Provincial Department of Finance, and the Shanxi Provincial Development and Reform Commission were major contributors to SEI policy promulgation.

**Table 5:** Top issuing bodies of SEI policies.

No.	Actor	Fre.	No.	Actor	Fre.
1	Shanxi Provincial People’s Government	33	6	Shanxi Provincial Department of Industry and Information Technology	5
2	Shanxi Provincial Committee of the Communist Party of China	10	7	Shanxi Provincial Department of Human Resources and Social Security	4
3	Shanxi Provincial Department of Finance	8	8	State-owned Assets Supervision and Administration Commission of Shanxi Provincial People’s Government	3
4	Shanxi Provincial Development and Reform Commission	7	9	Shanxi Provincial Department of Science and Technology	3
5	Shanxi Provincial Department of Education	6	–	–	–

Note: Partial list (frequency ≥ 3).

#### 4.1.2. Analysis of actor collaboration.

Inter-departmental relationships among government bodies influence SEI policy implementation, including content, promotion, and enforcement, as well as resource allocation, thereby reflecting local government attention allocation. Co-issuance practices reflect these interaction patterns. A collaboration network was constructed based on policy-making actors and their relationships using Gephi software (Table 6).

**Table 6:** Top institutions by network centrality.

No.	Institution	Betweenness centrality	No.	Institution	Degree centrality
1	Shanxi Provincial Development and Reform Commission	188.6667	1	Shanxi Provincial Development and Reform Commission	7
2	Shanxi Provincial Department of Commerce	104.1667	2	Shanxi Provincial Department of Finance	6
3	Shanxi Provincial Department of Ecology and Environment	90.0000	3	Shanxi Provincial Department of Education	4
4	Shanxi Provincial Bureau of Statistics	85.0000	4	Shanxi Provincial Department of Industry and Information Technology	4
5	Shanxi Provincial Copyright Administration	73.0000	5	Shanxi Provincial Department of Ecology and Environment	4

Note: The top 5 institutions in the collaboration ranking.

Table 6 shows an average degree centrality of 2.312 within the collaboration network. Textual analysis reveals that only 17 policies (22.83%) were co-issued by two or more entities. The Shanxi Provincial Development and Reform Commission (SPDRC), with a degree centrality of 7, maintains the closest ties with the Shanxi Provincial Department of Finance, the Department of Education, and the Department of Industry and Information Technology. Regarding betweenness centrality, the SPDRC ranked first (188.67), followed by the Department of Commerce and the Department of Ecology and Environment.

These metrics reveal specific coordination mechanisms. The SPDRC’s high degree centrality identifies it as the primary hub for inter-agency collaboration. However, the low average degree centrality (2.312) and low co-issuance rate (22.83%) suggest that coordination is largely dyadic and ad hoc rather than systemic. In practice, this structure may lead to information silos, where agencies communicate bilaterally but lack a multilateral platform for integrated policy design, resulting in overlapping or contradictory measures. The SPDRC’s high betweenness centrality (188.67) underscores its role as an indispensable broker; policies requiring input from both the Department of Finance and the Department of Ecology and Environment typically flow through it. While this brokerage ensures coordination, it also creates a bottleneck; the Commission’s limited administrative capacity may constrain cross-sectoral initiatives. In such broker-governed networks, decision-making can be rapid but lacks resilience; if the broker’s attention shifts, coordination stalls, and peripheral actors lack incentives to align actions.

In summary, synergy among SEI policy actors remains weak. The policy community exhibits a core-periphery structure characterized by the coexistence of high decentralization and centralization. While 71.67% of institutions issued policies independently—indicating strong departmentalism and resource fragmentation—55.81% of these issuances originated from the Shanxi Provincial People’s Government (SPPG). This concentration reinforces the SPPG’s absolute dominance but reflects a low willingness among other departments to collaborate, creating a “single-path” dependency. Collaborative groups are limited in scale (22.83%), and the network’s average degree centrality remains low (2.312).

### 4.2. Analysis of SEI policy instruments

#### 4.2.1. Demand-side policy instruments.

Demand-side instruments aim to generate market demand for SEI development, creating a market pull through mechanisms such as government procurement, application demonstrations, and consumer subsidies (Table 7). These instruments bridge the gap between innovation and industrialization. In Shanxi, demand-side instruments encompass four sub-categories: government subsidies (23.08%), government procurement (30.77%), application demonstrations (23.08%), and market expansion (23.08%). Government procurement holds the largest share, indicating a strategic reliance on public procurement to drive SEI development. The balanced distribution of the other three instruments reflects a comprehensive approach encompassing consumption incentives, technology validation, and channel expansion.

**Table 7:** Demand-side policy instruments for SEIs.

Type		Fre.	Percentage	Material example	Source
Demand-side policy	Market expansion	3	23.08**%**	“Deepen domestic and international cooperation....Targeting international market demand, establish export bases for SEIs, encourage large enterprises to go global and set up factories, and strive to occupy the commanding heights of international industrial competition in some advantageous fields.”	P32
	Application demonstration	3	23.08**%**	“Implement 100 pilot projects. Support projects that tackle key common technologies and the industrialization development of high-end products with a demonstrative and driving effect. Implement major industrial innovation and development projects,... Implement major application demonstration projects to promote a number of pilot projects and the results of major industrial innovation and development projects.”	P32
	Government subsidies	3	23.08**%**	“In accordance with provincial regulations, for the first purchase and first use of major technical equipment or major innovative products in the ‘first set’ category, the provincial finance department may provide a subsidy of 30% of the purchase price, not to exceed 1 million RMB.”	P48
	Government procurement	4	30.77**%**	“For our province’s ‘first set’ major technical equipment, ‘first edition’ software products, and ‘first batch’ raw materials that meet the government procurement catalog, explore the implementation of a government procurement system for first purchase and first use starting from January 1, 2019.”	P48

#### 4.2.2. Supply-side policy instruments.

Supply-side instruments primarily involve the provision of development factors of production, including financial funds, talent acquisition, technical support, and infrastructure construction (Table 8). While enterprises are the primary recipients of these resources, government intervention via supply-side instruments enhances allocation efficiency, thereby accelerating SEI development. Shanxi’s supply-side policies cover six categories: public services, technical support, spatial guarantees, talent security, financial support, and carriers and platforms. These are stratified into three tiers by frequency. The first tier is dominated by talent support (19), accounting for over one-third of supply-side instruments, highlighting government prioritization. The second tier includes public services (14), carriers and platforms (13), and financial support (9), accounting for 22.95%, 21.31%, and 14.75%, respectively, indicating systematic multi-area support. The third tier comprises technical support and spatial guarantees, totaling only 9.84% of supply-side instruments, reflecting their relative weakness in the current system.

**Table 8:** Supply-side policy instruments for SEIs.

Type		Fre.	Percentage	Material example	Source
Supply-side policy	Public services	14	22.95%	“Accelerate the construction of new-type infrastructure…”	P52
	Technical support	3	4.92%	“Address matters concerning frontier technology R&D, major key core technology breakthroughs, and application demonstrations that constrain the development of SEIs and public sectors in our province…”	P22
	Spatial guarantees	3	4.92%	“Relying on various development zones (parks) and guided by the layout of large backbone enterprises, accelerate the formation of a ‘one core, two belts, four plates’ spatial layout.”	P34
	Talent support	19	31.15%	“Vigorously support the introduction of high-level, precision, and cutting-edge talent in short supply. Focusing on the main economic front of our province, prioritize the introduction of urgently needed leading scientific and innovation talents and teams at international first-class and domestic top levels for our province…”	P25
	Financial support	9	14.75%	“Increase government financial support. Integrate existing policy resources and funding channels, make full use of national financial support and financial innovation policies, and establish a stable mechanism for government investment in the cultivation and development of SEIs…”	P2
	Carriers and platforms	13	21.31%	“Build a data traffic valley to promote the deep integration of the digital economy and the real economy…”	P52

#### 4.2.3. Environmental-side policy instruments.

Environmental-side instruments establish external conditions and institutional frameworks for industrial development, acting as catalysts through strategic planning, financial support, institutional innovation, and regulatory measures (Table 9). They build a sustainable industrial ecosystem and stimulate endogenous market dynamics, which is crucial for high-quality development. Although their impact is less immediate than supply-side instruments, it is more profound and fundamental. In Shanxi, strategic planning (27.50%), financial support (30.00%), and institutional innovation (22.50%) are the top three instruments. Together, they constitute 80% of environmental-side instruments, reflecting a focus on stimulating market vitality through macro-guidance, financial matching, and reform. Conversely, tax incentives, regulatory controls, and property rights protection receive limited attention; notably, property rights protection accounts for only 2.50%.

**Table 9:** Environmental-side policy instruments for SEIs.

Type		Fre.	Percentage	Material example	Source
Environmental-side policy	Strategic planning	11	27.50%	“Market access and competition mechanisms, technology standards, fiscal and tax incentives and investment and financing mechanisms, intellectual property protection policies, and talent team building for the development of SEIs will be more refined, cultivating about 15 first-class national-level enterprise technology innovation teams…”	P34
	Financial support	12	30.00%	“A failure tolerance rate of up to 30% of the investment amount for venture capital funds will be implemented,...”	P26
	Tax incentives	3	7.50%	“For the actual R&D expenses incurred by technology-based SMEs, if they are not deducted before tax at a 75% super-deduction rate when included in current period profits and losses, or if intangible assets formed are not amortized before tax at 175%…”	P48
	Regulations and controls	4	10.00%	“In the process of promoting the development of SEIs, implementing innovation projects, and advancing SOE reform, when problems arise in the performance of duties by responsible persons of government departments and SOEs, an objective and fair audit evaluation and handling should be made. If it falls under reasonable fault tolerance for reform and innovation, they will be exempted from liability.”	P26
	Property rights protection	9	22.50%	“For prefecture-level cities that vigorously cultivate and develop SEIs, have distinctive industrial advantages, strong technological innovation capabilities, and great development potential, priority support will be given for declaring the construction of national SEI clusters, encouraging qualified units to carry out institutional and mechanistic innovation…”	P17
	Institutional innovation	1	2.50%	“Encourage the convergence of intellectual property service resources towards SEIs and clusters of enterprises with distinctive advantages, helping enterprises achieve breakthroughs in key core technologies, apply for patents in core segments, plan domestic and international patent layouts, and register trademarks,…”	P40

Overall, the application of policy instruments in Shanxi’s SEI development exhibits significant structural disparities. As shown in Tables 7–9, demand-side instruments total 13 (11.40%), supply-side 61 (53.50%), and environmental-side 40 (35.10%). This indicates a policy landscape dominated by supply-side instruments, with environmental-side instruments serving as support and demand-side instruments remaining a significant weakness.

In summary, the SEI policy structure in this less-developed central region exhibits a “supply-environmental dual-drive” characteristic. The distribution confirms a heavy reliance on supply-side and environmental-side instruments, whereas demand-side instruments are underutilized. This pattern reflects a tendency to leverage supply-side instruments for rapid short-term industrial base construction, while relying on environmental-side instruments for long-term systemic guarantees. While this mix theoretically facilitates initial industry formation, its actual efficacy requires further verification through industrial performance data.

This policy preference stems from two primary factors. First, early-stage SEIs commonly face constraints such as immature technology, high costs, and low market recognition, prompting the government to prioritize supply-side instruments to achieve a “zero-to-one” breakthrough. Second, per path dependency theory, supply-side and environmental-side instruments align more closely with the government’s traditional policymaking pathways and administrative capacities than demand-side instruments; consequently, the former are adopted more readily due to lower design and implementation complexity.

### 4.3. Analysis of SEI policy themes

#### 4.3.1. Analysis of thematic domains.

High-frequency words were extracted from 60 SEI policy texts following word segmentation, denoising via stop word removal, and frequency calculation (Table 10). These keywords distill policy content and reveal structural relationships, thereby reflecting policy focus. Table 10 indicates that the most frequent terms are “development,” “manufacturing,” “Shanxi province,” “enterprise,” and “innovation,” suggesting that government initiatives center on provincial SEI development. Innovative development remains a consistent priority, with emphasis placed on manufacturing, enterprise incentives, technological innovation, and industrial clusters.

**Table 10:** Top 20 High-frequency words in policy texts.

No.	Keyword	Fre.	No.	Keyword	Fre.
1	strategic emerging industries	941	11	product	256
2	development	801	12	talent	185
3	manufacturing	621	13	system	185
4	shanxi province	499	14	research & development	184
5	enterprise	397	15	intelligent manufacturing	172
6	innovation	386	16	project	162
7	new materials	371	17	base	157
8	technology	340	18	application	150
9	high-end equipment	292	19	industry	145
10	equipment	277	20	production	137

#### 4.3.2. Analysis of topic structure.

Topic structure reflects core content and relative importance, indicating government attention allocation. Theme identification relied on topic-word distributions. Integrating coherence and perplexity metrics, the optimal number of topics was set to four, with a relevance parameter (λ) of 0.6 applied to extract semantically distinct terms. The top 10 keywords ranked by probability for each theme were extracted to form the thematic structure table (Table 11).

**Table 11:** Topic structure of SEI policies.

No.	Topic name		High-frequency words (Top 10)					
			No.	Keywords	Fre.	No.	Keywords	Fre.
1	Dual layout of industrial base and characteristics	Industrial base construction; Coal industry development	1	technology	0.024168	6	industry	0.014866
			2	enterprise	0.024227	7	product	0.013914
			3	equipment	0.016789	8	coalbed methane	0.011714
			4	cultivation	0.015178	9	project	0.009941
			5	base	0.014826	10	resource	0.009723
2	Intelligent upgrading	Hard-tech construction; Soft-tech empowerment	1	device	0.076534	6	equipment	0.030431
			2	materials	0.066738	7	high-performance	0.023127
			3	system	0.041610	8	software	0.019797
			4	service	0.044139	9	technology	0.017216
			5	intelligent	0.036168	10	internet	0.016945
3	Talent echelon construction	High-level talent development; Talent cultivation; Innovation team construction	1	talent	0.048959	6	team	0.015759
			2	information	0.025033	7	professional	0.013397
			3	leading	0.024941	8	cultivation	0.013974
			4	government	0.019579	9	standardization	0.012524
			5	project	0.018182	10	entrepreneurship	0.012137
4	Institutional reform and cluster ecosystem cultivation	Industrial cluster development; Institutional mechanism reform	1	enterprise	0.095391	6	economy	0.016122
			2	cluster	0.029945	7	service	0.015805
			3	reform	0.018089	8	industry	0.014866
			4	investment	0.016742	9	management	0.014599
			5	transformation	0.016522	10	product	0.013648

Note: Keep to 6 decimal places.

Topic-word distributions reveal latent topic structures, where higher probability values indicate stronger word-topic associations. As shown in Table 11, SEI development policies primarily involve four topics: the dual layout of industrial base and characteristics, intelligent upgrading, talent echelon construction, and institutional reform with cluster ecosystem cultivation.

(1) Dual layout of industrial base and characteristics

Dominated by “technology” (0.0242) and “enterprise” (0.0242), this theme includes keywords such as “equipment” (0.0168), “cultivation” (0.0152), and “base” (0.0148), indicating a policy focus on fostering innovation entities and infrastructure. Notably, terms like “coalbed methane” (0.0117) and “resource” (0.0097) reflect Shanxi’s strategy to leverage local endowments for comparative advantage. For instance, Shanxi promotes the modern coal chemical industry; in the coalbed methane sector, it advances exploration in the Qinshui and Hedong coalfields, trials surface development and underground drainage, and coordinates the integrated utilization of coalbed methane, natural gas, coke oven gas, and coal-to-gas alongside provincial pipeline networks [36].

(2) Intelligent upgrading

Centered on “equipment” (0.0765) and “materials” (0.0667), this theme highlights the hardware foundation for industrial upgrading. Keywords such as “system” (0.0416), “service” (0.0441), “intelligent” (0.0362), “software” (0.0198), and “internet” (0.0169) constitute the technical architecture for digital transformation. Shanxi supports high-end, intelligent, and green manufacturing transitions, focusing on intelligent coal equipment. Policies encourage industrial internet platforms to stabilize employment and drive transformation, promoting the deep integration of producer services with manufacturing. Furthermore, the government champions digital demonstration projects and AI applications to build an ecosystem where digital technology and the real economy merge.

(3) Talent echelon construction

With “talent” (0.0490) as the core element, this theme underscores the fundamental role of human capital. Terms like “leading” (0.0249) and “team” (0.0158) reflect a hierarchical approach prioritizing top-tier leadership and collaborative teams. “Entrepreneurship” (0.0121) and “cultivation” (0.0140) form a full-chain support system covering introduction and development. Shanxi has issued specific policies, such as the *Implementation Opinions on the Cultivation Project for Leading Talents in Emerging Industries* [37] and *the Measures for Supporting the Introduction of High-Level, Precision, and Cutting-Edge Talent in Short Supply (for Trial Implementation)* [38], establishing a sustainable talent support system through systematic selection, funding, and management processes.

(4) Institutional reform and cluster ecosystem cultivation

“Enterprise” (0.0954) predominates in this theme, reaffirming the principal role of firms in high-quality SEI development. “Cluster” (0.0299) defines the organizational path, while “reform” (0.0181), “investment” (0.0167), and “management” (0.0146) relate to governance modernization. This indicates a systemic shift from dispersed development to efficient clustering. Shanxi reinforces enterprises’ roles in industrial chains while deepening management reform. Financially, it leverages government-guided funds and subsidies to allocate resources to regional specialty industries, fostering integrated development across SEI clusters [39].

#### 4.3.3. Analysis of topic intensity.

**Fig 4:**
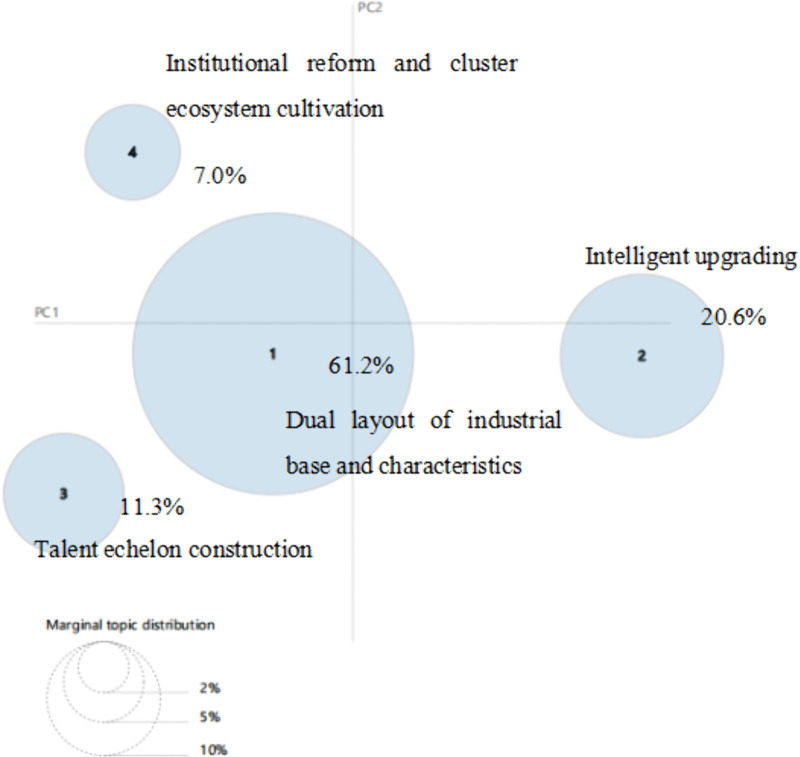
Topic proportions of SEI policies (Source: Generated by the author using Python).

Topic proportion serves as a key indicator of government attention allocation. Defined as the relative proportion of a topic within SEI policy documents, it quantifies the government’s inclination toward specific thematic areas, where a higher intensity signaling greater strategic priority. Based on LDA topic mining results, the topic intensity of SEI policies was calculated and visualized (Fig 4).

As shown in Fig 4, the distribution of topic proportions is highly skewed. “Dual layout of industrial base and characteristics” constitutes the dominant proportion at 61.2%, indicating a high concentration of resources on strengthening the industrial base and cultivating market entities. “Intelligent upgrading” accounts for the second-highest proportion at 20.6%, demonstrating the government’s reliance on intelligent technologies to drive industrial transformation. Meanwhile, “talent echelon construction” and “institutional reform and cluster ecosystem cultivation” represent 11.3% and 7.0%, respectively. This reflects a strategy to support sustainable development through two distinct dimensions: talent elements and institutional arrangements.

In summary, Table 12 integrates the preceding findings. Aligned with the three-dimensional analytical framework depicted in Fig 1, the table shows that distinct policy themes correspond to distinct combinations of policy actors and policy instruments. This integration empirically demonstrates that government policy attention is operationalized through an actor-instrument-theme configuration, thereby validating the proposed analytical framework.

**Table 12:** Integrative Actor–Instrument–Theme of SEI policies.

Policy actors		Policy instrument		Policy theme
betweenness centrality	Shanxi Provincial Development and Reform Commission	Demand-side policy	Market expansion; application demonstration; government subsidies; government procurement	Dual layout of industrial base and characteristics
	Shanxi Provincial Department of Commerce	Supply-side policy	Public services; technical support; spatial guarantees; talent support; financial support; carriers and platforms	Intelligent upgrading
degree centrality	Shanxi Provincial Development and Reform Commission	Environmental-side policy	Strategic planning; financial support; tax incentives; regulations and controls; property rights protection; institutional innovation	Talent echelon construction
	Shanxi Provincial Department of Finance	–	–	Institutional reform and cluster ecosystem cultivation

Note: The top-ranked institutions by betweenness and degree centrality.

## 5. Discussion and conclusion

### 5.1. Discussion

This discussion interprets the empirical patterns observed in Shanxi’s policy documents. Causal language is employed solely to articulate potential mechanisms or plausible risks rather than to assert verified causal relationships. Due to the absence of outcome data, these interpretations remain hypothetical and warrant future empirical validation.

First, policy-making bodies for Strategic Emerging Industries (SEIs) in less-developed central regions exhibit weak synergy. The SEI policy network features a loose structure characterized by a preference for individual action over collaboration. Specifically, 71.67% of institutions issued policies independently, with 55.81% of these concentrated in the Shanxi Provincial People’s Government. While this centralization reinforces decision-making dominance and enforcement intensity, it engenders single-path dependence, as other departments demonstrate limited willingness to collaborate proactively. Collaborative groups account for merely 22.83% of total issuances, resulting in a sparse overall network with an average centrality of 2.312. This weak synergy potentially constrains the integration efficiency and systemic effectiveness of policy resources. Network theory posits that low density coupled with high centralization undermines a system’s capacity to address complex, multi-faceted problems requiring simultaneous inputs from finance, technology, land, and environmental regulation. In Shanxi, the bottleneck effect imposed by the Development and Reform Commission may explain the underdevelopment of demand-side instruments, which necessitate multi-agency and market coordination, relative to supply-side instruments implementable by a single agency. The observed core-periphery structure, characterized by high centralization and low horizontal density, entails dual implications. Centralized control ensures rapid policy rollout and political accountability, potentially advantageous in less-developed central regions with limited administrative capacity. Conversely, such structures are vulnerable to coordination failures regarding cross-sectoral issues, such as aligning fiscal incentives with land-use planning. Whether Shanxi will experience these adverse consequences remains an empirical question, yet the structural pattern itself warrants concern.

Second, SEI policy allocation in less-developed central regions relies on a supply-side and environmental-side dual-engine model. The study reveals a significant imbalance: supply-side instruments account for 53.50%, environmental-side for 35.10%, and demand-side for merely 11.40%. This configuration represents a rational choice under the dual constraints of resource endowments and administrative capacity. In their early stages, SEIs face immature technologies, high costs, and low market recognition, prompting governments to prioritize supply-side support to achieve initial breakthroughs. The supply-side and environmental-side dominant model is arguably efficient for short-to-medium-term industrial takeoff, as supply-side tools directly alleviate shortages of capital, talent, and infrastructure, while environmental-side tools establish a stable regulatory framework. In Shanxi, this model has likely facilitated the construction of basic SEI infrastructure, such as new material parks and high-end equipment zones, and attracted initial investment. However, this model becomes increasingly detrimental as SEIs progress toward commercialization and scaling. Without demand-side tools like government procurement and application demonstrations, innovative products may fail to gain market traction. Furthermore, this allocation logic reflects substantial inertia from traditional policy-making pathways, as supply-side and environmental-side instruments align more closely with existing administrative capacities and risk appetites. Three long-term risks are identified: first, supply-side bias may encourage corporate rent-seeking over market-driven innovation due to weak demand-side pull; second, environmental-side instruments may inadvertently favor incumbent state-owned enterprises, crowding out smaller private entrants; third, path dependency locks in administrative routines, hindering the subsequent development of demand-side tools.

Third, governmental attention allocation to SEI policy themes in less-developed central regions exhibits a pyramid-shaped structure. Attention intensity across policy themes demonstrates a clear gradient: “Dual layout of industrial base and characteristics” dominates at 61.2%, followed sequentially by “Intelligent upgrading” (20.6%), “Talent echelon construction” (11.3%), and “Institutional reform and cluster ecosystem cultivation” (7.0%). This differentiated allocation, strengthening foundations, promoting upgrading, and providing support, highlights the government’s rational strategic prioritization given the weak SEI industrial base. Unlike developed regions that pursue cutting-edge technological breakthroughs, governments in less-developed central regions primarily act as infrastructure providers. They concentrate on constructing industrial foundations and cultivating distinctive features to compensate for market mechanism deficiencies and establish prerequisites for industrial germination. In Shanxi, this allocation likely reflects a progressive development logic shaped by the region’s developmental stage and resource-dependent history. Whether this pattern generalizes to other less-developed central regions warrants further comparative study.

These observed patterns can be interpreted through several theoretical lenses. First, the fragmented collaboration network (71.67% independent issuances) aligns with the holistic governance literature, which posits that departmental silos and departmentalism impede coordinated policy implementation [40]. However, the findings also reveal a countervailing force: the provincial government’s strong centralizing role (55.81% of independent issuances) suggests a “centralized fragmentation” model, wherein authority is concentrated in a single actor rather than diffused through horizontal collaboration. This hybrid pattern of high centralization and low horizontal synergy may be a distinctive feature of less-developed, resource-dependent regions where administrative capacity is concentrated in a few core agencies. Second, the dominance of supply-side and environmental-side instruments (88.6% combined) over demand-side tools (11.4%) can be understood through path dependence theory [41]. Governments in less-developed central regions have historically relied on direct factor provision (supply) and regulatory guidance (environment) to drive industrial development—a repertoire inherited from the planned economy era. Demand-side instruments, requiring sophisticated market coordination and private-sector engagement, represent a departure from this administrative routine. The observed imbalance thus reflects institutional inertia alongside rational choice. This study contributes to the path dependency literature by elucidating how policy instrument portfolios become locked in through repeated use, even when demand-side tools are necessary for long-term sustainability. Third, the pyramid-shaped thematic attention, with foundational themes at 61.2%, talent pipeline at 11.3%, and institutional reform at 7.0%, resonates with punctuated equilibrium theory in agenda-setting [42]. In less-developed central regions, industrial policy attention may be punctuated by a single overriding priority (foundation building), while other dimensions receive residual attention. This case suggests that the foundations-first pattern constitutes a distinct equilibrium state for resource-dependent latecomer regions, carrying implications for the timing and mechanisms of policy attention re-balancing.

### 5.2. Implications for public policy

This study yields several policy implications for SEI development in less-developed central regions. Derived from observed attention allocation patterns in Shanxi province, these implications represent potential risk-mitigation strategies based on analytical reasoning rather than evidence-based prescriptions. Policymakers in analogous regions should evaluate the transferability of these suggestions within their local contexts.

First, enhance policy synergy for SEIs. The current policy network is fragmented, with issuing bodies operating in isolation, thereby undermining policy performance. To address these deficits, excessive centralization should be reduced by cultivating multi-hub coordination. Instead of relying predominantly on the Development and Reform Commission for cross-agency links, the provincial government should designate two or three additional core departments—such as the Department of Science and Technology and the Department of Industry and Information Technology—as co-coordinators for specific SEI sub-sectors. This distributes the coordination burden and mitigates bottleneck risks. Furthermore, to counteract the low collaboration density (only 22.83% of policies are co-issued), regular cross-departmental consultation should be institutionalized. This entails mandatory quarterly inter-agency working meetings chaired by a rotating department, with fixed agendas to review SEI progress, resolve overlapping mandates, and align policy instruments, thereby ensuring that minutes and action items are systematically tracked. Finally, information silos resulting from absent real-time data sharing can be mitigated by establishing a streamlined digital platform. A shared dashboard listing all SEI policies under development, their lead agencies, and their status would reduce duplicated efforts and conflicting measures without necessitating complex organizational restructuring.

Second, optimize the structure of SEI policy instruments. Given the significant imbalance in the current instrument portfolios, a phased optimization strategy is recommended over uniform expansion. On the supply side, whereas talent assurance, public services, and platform construction are adequately addressed, financial, technological, and spatial support remains inadequate. Governments should shift from universal subsidies to an ex-post reward mechanism predicated on key technological breakthroughs and innovation performance. Additionally, full-cycle project evaluations with dynamic exit mechanisms should be established to ensure that public resources genuinely cultivate market-competitive enterprises. On the environmental side, efforts must continue to focus on standardization, fiscal reform, and intellectual property protection to reduce institutional transaction costs and foster a stable, predictable innovation environment. Strengthening intellectual property rights is particularly crucial for integrating local enterprises into global innovation and value chains. On the demand side—the weakest component—governments should pilot demand-side instruments prior to full industrial maturation. Specific measures include expanding government procurement by incorporating more strategic emerging products into procurement catalogs, implementing a government first-purchase system to overcome initial deployment bottlenecks for innovative products, and broadening application demonstration projects beyond technology validation to integrate business models and application scenarios. These steps would effectively bridge the innovation and market ecosystems.

Third, rebalance resource allocation for SEI development. The pyramid-shaped factor allocation in less-developed central regions facilitates rapid industrial base formation but neglects critical long-term drivers such as talent, institutional quality, and market demand. To sustain industrial momentum while preserving existing strengths, a phased transition strategy is required. The prevailing supply-side and environmental-side dual-engine model, while appropriate for the early take-off phase, risks creating enduring distortions as industries mature. Policymakers should thus plan a gradual rebalancing rather than an abrupt policy shift. Specifically, a three-phase roadmap is proposed. Phase I (0–5 years), foundation building, this phase maintains strong supply-side support while piloting demand-side instruments in selected sectors. Phase II (5–10 years), gradual rebalancing, this phase shifts supply-side subsidies toward performance-based rewards, expands government procurement for SEI products, and introduces independent subsidy evaluations. Phase III (10 + years), market-driven innovation, this phases out direct subsidies, scales up demand-side instruments, and delegates coordination functions to industry associations and intermediaries. Transitions between phases should be triggered by objective indicators—such as the SEI share of GDP, patent commercialization rate, and SME participation rate—rather than fixed calendar years.

### 5.3. Conclusion

Drawing on 60 policy texts from Shanxi province, this study employs a case study, LDA topic modeling, and SNA to examine government attention allocation for SEIs. It reveals a foundation-first, gradient-layout pattern characterized by fragmented actor networks, supply-side and environmental-side-dominated instruments, and pyramid-shaped thematic attention. These patterns are most directly applicable to regions with similar resource dependence and early developmental stages; generalization to non-resource-based, less-developed central regions or advanced regions requires further empirical testing.

The core conclusions are threefold. First, the policy-making structure for SEIs exhibits weak synergy among government actors. The policy network demonstrates a loose structure with pronounced single-path dependence, where a majority of institutions act independently, spearheaded by the provincial government. This fragmentation inherently constrains the integration efficiency and systemic effectiveness of policy resources. Second, the policy instrument configuration demonstrates a distinct supply-side and environmental-side dual-engine model. A significant imbalance exists, with an overwhelming emphasis on supply-side (53.50%) and environmental-side (35.10%) instruments, while demand-side policies are severely underrepresented (11.40%). This pattern aligns with rational choice under resource and administrative constraints and may reflect path dependence; however, these interpretations require comparative testing. Third, the thematic allocation of government attention exhibits a pyramid-shaped structure. Attention is heavily skewed toward foundational development, with “Industrial base and characteristic layout” dominating at 61.2%, whereas themes related to upgrading, talent pipeline development, and institutional reform receive progressively less attention. This reveals a pragmatic “infrastructure-provider” strategy, wherein the government prioritizes establishing a solid industrial base before pursuing advanced, innovation-driven growth.

#### 5.3.1. Contributions of the paper.

This study makes two theoretical contributions. First, it integrates the ABV with policy instrument theory into a unified Actor–Instrument–Theme framework for analyzing government attention allocation. While ABV has been applied in organizational and strategic management research, its operationalization in public policy, particularly through the joint analysis of policy actors, instruments, and thematic priorities, remains underdeveloped. This framework offers an analytical template adaptable to other policy domains (e.g., environmental regulation and innovation policy) and regional contexts. Second, the study proposes the concept of the “foundation-first, gradient-layout” model as an ideal-type for attention allocation mechanisms in resource-dependent, less-developed central regions. This model posits that in contexts with weak industrial bases and concentrated administrative capacity, governments rationally prioritize foundational elements (infrastructure, enterprises, and basic technology) before gradually shifting attention to upgrading, talent pipelines, and institutional reforms. Regarding empirical contributions, this study provides the first systematic, multi-dimensional mapping of government attention in a less-developed central region of China using LDA and SNA. It documents three interrelated features defining the “foundation-first” model: fragmented yet centralized actor networks, supply-side and environmental-side dominated instrument portfolios, and pyramid-shaped thematic attention. These findings establish a baseline for future longitudinal and comparative studies.

#### 5.3.2. Limitations and future prospects.

Notwithstanding the aforementioned contributions, this study possesses inherent limitations that delineate avenues for future research. First, this study is confined to a single province. Although Shanxi represents a typical resource-dependent, less-developed central region, the findings remain context-specific; future research should conduct multi-case comparative studies to establish boundary conditions. Second, the theoretical propositions, such as the “foundation-first” ideal type, are derived from a single case and should be treated as hypotheses for future testing rather than confirmed empirical generalizations. Third, the study employs a cross-sectional design with policies aggregated from 2010 to 2025. As attention allocation may shift across different SEI developmental phases, longitudinal designs are needed to trace attention evolution. Despite these limitations, the study provides an empirically grounded, multi-dimensional description of government attention in an understudied context, serving as a hypothesis-generating foundation for future research.

## References

[1] Zhai JG, Li JW, Zhao D, Zhao XZ. The spatial interaction mechanism of geo-network agglomeration in strategic emerging industries: A case study of Liuhe District, Nanjing. Geogr Res. 2025;44(9):2534–53.

[2] Xu CW, Shi YL. The level of collaborative innovation development, regional differences, and spatial convergence characteristics of strategic emerging industries. Stat Decis. 2025;41(9):134–8. doi: 10.19445/j.cnki.10.13546/j.cnki.tjyjc.2025.09.023

[3] Gertler MS, Oinas P, Storper M, Scranton P. Discussion of regional advantage: culture and competition in Silicon Valley and Route 128 by Anna Lee Saxenian. Econ Geogr. 1995;71(2):199–207. doi: 10.2307/144358

[4] Cooke P. Biotechnology clusters as regional, sectoral innovation systems. Int Reg Sci Rev. 2002;25(1):8–37. doi: 10.1177/016001760202500102

[5] Popkova EG, Ragulina YV, Bogoviz AV. Industry 4.0: Industrial revolution of the 21st century. Cham: Springer; 2019. doi: 10.1007/978-3-319-94310-7

[6] Ji P, Yuan LL. Spatial evolution characteristics and optimization strategies of strategic emerging industries in the Beijing-Tianjin-Hebei region. Hebei Acad J. 2025;45(6):103–9.

[7] Zhao L, Sun CY. Spatial agglomeration and co-agglomeration patterns of strategic emerging industries in China. Areal Res Dev. 2025;44(4):18–25.

[8] Yao W, Hu SS, Chu ZW. Research on the policy instrument system of China’s provincial strategic emerging industries: based on statistical analysis of policy indices. Sci Technol Manage Res. 2020;40(7):26–34.

[9] Nishimura J, Okamuro H. Subsidy and networking: the effects of direct and indirect support programs of the cluster policy. Res Policy. 2011;40(5):714–27. doi: 10.1016/j.respol.2011.01.011

[10] Xie XY, Wang XL. The evolution and content evaluation of China’s 5G strategic emerging industry policies. Sci Manage Res. 2024;42(3):45–52. doi: 10.19445/j.cnki.15-1103/g3.2024.03.006

[11] Tang W, Qiu X, Chen ZX. Government guidance funds and the development of strategic emerging industries: From the perspective of government-market collaboration in industrial development. Quant Econ Technol Econ Res. 2025;42(9):52–71. doi: 10.13653/j.cnki.jqte.20250724.001

[12] Yao SJ, Dong ZM. Research on the path of artificial intelligence enabling new quality productive forces in strategic emerging industries. J Zhejiang Gongshang Univ. 2025;1:100–13. doi: 10.14134/j.cnki.cn33-1337/c.2025.01.010

[13] Cattaneo MC, Mazzucato M. The entrepreneurial state. Debunking public vs. private sector myths. Econ Polit. 2013;31(1):103–10. doi: 10.1428/76495

[14] Cantner U, Kösters S. Picking the winner? Empirical evidence on the targeting of R&D subsidies to start-ups. Small Bus Econ. 2011;39(4):921–36. doi: 10.1007/s11187-011-9340-9

[15] Zeng RX, Mo ML. The measurement of government attention: approaches, content, and prospects. J Huazhong Univ Sci Technol (Soc Sci Ed). 2023;37(1):66–73. doi: 10.19648/j.cnki.jhustss1980.2023.01.09

[16] Mintrom M. Herbert A. Simon, Administrative Behavior: A Study of Decision-Making Processes in Administrative Organization. In: Lodge M, Page EC, Balla SJ, editors. The Oxford Handbook of Classics in Public Policy and Administration. Oxford: Oxford University Press; 2015. doi: 10.1093/oxfordhb/9780199646135.013.22

[17] Berkhout J. The politics of attention: how government prioritizes problems. Acta Polit. 2008;43(4):504–7. doi: 10.1057/ap.2008.26

[18] Ocasio W. Towards an attention-based view of the firm. Strateg Manage J. 1997;18(S1):187–206. doi: 10.1002/(SICI)1097-0266(199707)18:1<187::AID-SMJ936>3.0.CO;2-K

[19] Dai K. Attention allocation: A new perspective for studying government behavior. Theory Mon. 2017;3:1–16. doi: 10.14180/j.cnki.1004-0544.2017.03.019

[20] Rothwell R. Reindustrialization and technology: towards a national policy framework. Sci Public Policy. 1985;12(3):113–30. doi: 10.1093/spp/12.3.113

[21] Rogge KS, Reichardt K. Policy mixes for sustainability transitions: an extended concept and framework for analysis. Research Policy. 2016;45(8):1620–35. doi: 10.1016/j.respol.2016.04.004

[22] Wang ZB. The landscape overview and critical review of domestic case study methods. Manage Case Stud Rev. 2022;15(3):335–46.

[23] Huang ZH. Differences and progression arrangements between multiple-case and single-case studies: theoretical discussion and case analysis. Manage Case Stud Rev. 2010;3(2):183–8.

[24] Eisenhardt KM. Building theories from case study research. Acad Manage Rev. 1989;14(4):532–50. doi: 10.5465/amr.1989.4308385

[25] Blei DM, Ng AY, Jordan MI. Latent Dirichlet allocation. J Mach Learn Res. 2003;3:993–1022. doi: 10.1145/2615569.2615680

[26] Wang L. Typical social network analysis software tools and analytical methods. China Educ Technol. 2009;4:95–100.

[27] Hu AG. Research on development issues in underdeveloped regions. Reform. 1994;3:110–7.

[28] Si LJ. Development in China’s underdeveloped regions: new issues, new challenges, and new paths. J Wuhan Univ (Philos Soc Sci Ed). 2025;78(5):129–40. doi: 10.14086/j.cnki.wujss.2025.05.013

[29] Hassink R, Isaksen A, Trippl M. Towards a comprehensive understanding of new regional industrial path development. Reg Stud. 2019;53(11):1636–45. doi: 10.1080/00343404.2019.1566704

[30] Barberá P, Boydstun AE, Linn S, McMahon R, Nagler J. Automated text classification of news articles: a practical guide. Polit Anal. 2021;29(1):19–42. doi: 10.1017/pan.2020.8

[31] Yang S, Keller FB, Zheng L. Social network analysis: methods and applications. Beijing: Social Sciences Academic Press; 2019.

[32] Griffiths TL, Steyvers M. Finding scientific topics. Proc Natl Acad Sci U S A. 2004;101 Suppl 1(Suppl 1):5228–35. doi: 10.1073/pnas.0307752101 14872004 PMC387300

[33] Guan P, Wang YF. Research on methods for determining the optimal number of topics in LDA topic modeling for scientific and technical information analysis. Mod Libr Inf Technol. 2016;9:42–50.

[34] Wang JR, Chen Z. A comparative study on text topic extraction based on latent Dirichlet allocation. Inf Sci. 2018;36(1):102–7. doi: 10.13833/j.issn.1007-7634.2018.01.018

[35] Nikolenko SI, Koltcov S, Koltsova O. Topic modelling for qualitative studies. J Inf Sci. 2017;43(1):88–102. doi: 10.1177/0165551515617393

[36] Shanxi Provincial People’s Government. Opinions on Accelerating the Cultivation and Development of Strategic Emerging Industries. 2011 [cited 2026 May 27]. Available from: http://www.shanxi.gov.cn/zfxxgk/zfxxgkzl/fdzdgknr/lzyj/szfwj/202205/t20220513_5975896.shtml

[37] Shanxi Provincial Department of Human Resources and Social Security. Notice on Issuing the Implementation Opinions for the Cultivation Project of Leading Talents in Emerging Industries in Shanxi Province. 2011 [cited 2026 May 27]. Available from: http://rst.shanxi.gov.cn/zwyw/zcfg/bmwj/201206/t20120604_66875.html

[38] Shanxi Provincial Department of Finance. Measures for Supporting the Introduction of High-level, Precision and Urgently Needed Talents (Trial). 2012 [cited 2026 May 27]. Available from: https://czxx.taiyuan.gov.cn/czfg/20171130/180431.html

[39] Shanxi Provincial Development and Reform Commission. Notice of the Shanxi Provincial Development and Reform Commission on Organizing the Application for Provincial-level Strategic Emerging Industry Integrated Clusters. 2017 [cited 2026 May 27]. Available from: http://fgw.shanxi.gov.cn/sxfgwzwgk/sxsfgwxxgk/xxgkml/zfxxgkxgwj/202304/t20230410_8327773.shtml

[40] Leat D, Setzler K. Towards holistic governance: The new reform agenda. Basingstoke: Palgrave; 2002.

[41] Setterfield M. Path dependency. Routledge handbook of macroeconomic methodology. London: Routledge; 2023.

[42] Givel M. The evolution of the theoretical foundations of punctuated equilibrium theory in public policy. Rev Policy Res. 2010;27(2):187–98. doi: 10.1111/j.1541-1338.2009.00437.x

